# The Microalgae *Phaeodactylum tricornutum* Is Well Suited as a Food with Positive Effects on the Intestinal Microbiota and the Generation of SCFA: Results from a Pre-Clinical Study

**DOI:** 10.3390/nu14122504

**Published:** 2022-06-16

**Authors:** Lena Stiefvatter, Ulrike Neumann, Andreas Rings, Konstantin Frick, Ulrike Schmid-Staiger, Stephan C. Bischoff

**Affiliations:** 1 Institute of Clinical Nutrition, University of Hohenheim, Fruwirthstr. 12, 70593 Stuttgart, Germany; lena.stiefvatter@uni-hohenheim.de (L.S.); ulrike.ne@web.de (U.N.); andreas.rings@uni-hohenheim.de (A.R.); 2 Institute of Interfacial Process Engineering and Plasma Technology, University of Stuttgart, 70569 Stuttgart, Germany; konstantin.frick@igvp.uni-stuttgart.de; 3 Fraunhofer Institute for Interfacial Engineering and Biotechnology, 70569 Stuttgart, Germany; ulrike.schmid-staiger@igb.fraunhofer.de

**Keywords:** *Phaeodactylum tricornutum*, eicosapentaenoic acid, fucoxanthin, β-glucan, chrysolaminarin

## Abstract

Microalgae such as *Phaeodactylum tricornutum* (PT) are a sustainable source of nutrients, especially eicosapentaenoic acid (EPA), fucoxanthin (Fx), and chrysolaminarin (Chrl), the concentrations of which can vary depending on the culture conditions. We generated three types of diets containing either an EPA- and Fx-rich (EPA/Fx) or Chrl-rich microalgae (with 5, 15, or 25% added to the diet) or an isocaloric control diet (CD). These diets were evaluated over 14 days in young C57BL/6J mice for safety and bioavailability, short-chain fatty acid (SCFA) production, and microbiome analysis. Both microalgae diets increased body weight gain dose-dependently compared to the CD. Microalgae-derived EPA was well absorbed, resulting in increased liver and fat tissue levels and a decrease in the n-6:n-3 ratio in liver tissue. Both microalgae diets increased the production of selected SCFA and decreased the Firmicutes/Bacteriodota ratio, whereas the Chrl-rich diet led to an increase in *Akkermansia*. Doses of up to 4621 mg Chrl, 920 mg EPA, and 231 mg Fx per kg body weight daily were tolerated without adverse effects. This pre-clinical study shows that PT is suitable for mouse feed, with positive effects on microbiota composition and SCFA production, suggesting beneficial effects on gut health.

## 1. Introduction

Climate change will alter the quantity and quality of food. Consumption and production patterns need to be adapted in a sustainable way to achieve the 2030 sustainable development goals. One aim is the sustainable use of the oceans [1]; therefore, a change in fishing practice needs to be undertaken. Microalgae could be used as an alternative raw material; they can be grown in bioreactors without farmland and provide a nutrient-rich alternative to high-value compounds, such as proteins and fatty acids [2]. In particular, the diatom PT seems to be interesting for human nutrition. It is a unicellular microalga living in water and containing high amounts of protein, the carotenoid Fx, EPA, an omega-3 polyunsaturated fatty acid (n-3 PUFA), and Chrl, a β-glucan [3]. It can be cultivated at different salt concentrations (marine, brackish, and even lower (<10 gNaCl L^−1^). Under nutrient-repleted growth conditions, PT generates large amounts of Fx and EPA.

Fx is synthesized by photosynthetic organisms for photoprotection and light harvesting and belongs to the xanthophylls. It is a significant carotenoid in brown (micro)algae and seaweeds [4,5]. Fx accumulation in the liver and adipose tissue [6] in mice was previously demonstrated, as well as metabolization and safe intake in humans [7]. Due to its antioxidant and anti-inflammatory properties [8], as well as anti-obesity effects [9], Fx is already being investigated for health benefits [10]. Microalgae are generally the primary producers of EPA and docosahexaenoic acid (DHA). However, the microalga PT, which was used in this study, mainly produces EPA [11]. Its biomass composition varies according to species and environmental factors [12]. EPA and DHA are important for human nutrition because the essential fatty acid (FA) α-linolenic acid (ALA) is insufficiently converted into EPA and DHA [13]. The direct consumption of 250–300 mg EPA+DHA daily or consumption of fish one to two times per week is recommended [14], as the immune mediators produced from DHA and EPA shift the balance towards anti-inflammation [15]. Because fish only acquire n-3 PUFAs by eating microalgae, the direct consumption of microalgae is a sustainable alternative [16]. *Schizochytrium* sp. and *Ulkenia* sp. oils are already approved as DHA sources for human consumption. The microalga *Odontella aurita*, rich in EPA, is also approved as whole microalga but only in small amounts (0.5–1.5%) for flavoring [17]. For health benefits, more significant amounts of microalgal biomass in human nutrition need to be investigated. PT is not yet approved as a whole microalga, although its nutritional composition is similar to *Odontella aurita* [18]. In the USA, an EPA-rich oil from PT is already on the market; in Europe, the product is still in the approval process by the European Food Safety Authority (EFSA). Eating this microalga after cell disruption by a ball mill has already been shown to result in good bioavailability of EPA and accumulation in mouse liver tissue [19] and human plasma [7]. A further component of PT biomass is Chrl, a water-soluble β-(1,3)/β-(1,6 β−glucan (11:1) that is accumulated from PT under nutrient-depleted cultivation conditions [11]. It serves as primary energy and carbon storage and is dissolved in the cytosol [20]. Macroalgae, such as brown algae of the genera *Laminaria* and *Saccharina*, possess β-glucan laminarin, which is very similar to Chrl, with a β-(1,3)/β-(1,6) glucan structure (15:1) [21], and can be used in animal feed [22]. Other sources of β-glucans include grain, fungi, bacteria, and yeast [23]. They are already used in foods, medicine, and cosmetics [24] due to nutraceutical functions, such as antitumor, anti-inflammatory, antioxidant, anticoagulant, and immunostimulant effects [23,25]. Similar properties have already been reported for laminarin [26], Chrl [27,28], and Chrl from PT [29,30]. Due to the various ingredients of PT and their health-promoting properties, it is suspected to promote intestinal health. These include the formation of short-chain fatty acids (SCFA), the promotion of SCFA-producing bacteria, and strengthening of the intestinal barrier [31,32,33].

The current study evaluates the difference and safety of two different biomass samples of the microalga PT. An EPA- and Fx-rich (EPA/Fx) and Chrl-rich biomasses (Chrl-rich) were added at doses of 5%, 15%, and 25% to the feed of adult female C57BL/6 mice for 14 days and compared to a PT-free control diet (CD). Diets were evaluated for safety aspects, such as feed consumption, energy in feces, fatty acid content, and gut health benefits, such as gut intestinal permeability. Therefore, the relative expression of zonula occludens protein-1 (ZO1), which activates the tight junctions (TJ) and occludin, a tight junction protein of the intestinal barrels that keep the TJ closed, are measured in the ileum. In feces, SCFA and the gut microbiome were analyzed by 16S ribosomal RNA sequencing.

## 2. Materials and Methods

### 2.1. Phaeodactylum Tricornutum Culture and Experimental Diets

In this study, PT biomass was used from the strain PT SAG 1090-1b. This biomass was produced at the Fraunhofer CBP (Leuna, Saxony-Anhalt, Germany) using 180 L flat-panel airlift (Subitec GmbH, Stuttgart, Germany) reactors under outdoor conditions. Two batches of the biomass (Fx/EPA-rich and Chrl-rich) were produced under cultivation conditions. The EPA/Fx-rich biomass was produced under nutrient-repleted conditions. In contrast, the Chrl-rich biomass was cultivated under nitrogen-depleted conditions for several days before the harvest. After the harvest, both batches of biomass were treated the same. First, the biomass was concentrated to approximately 250 g L^−1^ via centrifugation (Clara 20, Alfa Laval, Glinde, Germany) and stored at −20 °C until further processing. After thawing, the biomass was diluted with deionized water to 100 g L^−1^. Subsequently, the cells were disrupted in a ball mill (PML-2, Bühler AG, Uzwil, Switzerland) according to Derwenskus et al. [34], freeze-dried, and pulverized as described previously [7]. The feed manufacturer, sSniff Spezialdiäten GmbH (ssniff Spezialdiäten GmbH, Soest, Germany) added both versions of PT at concentrations of 5%, 15%, and 2% (Table 1) to the diets, which were isocaloric and isoproteinogenic to the CD. The Fx content in chow was measured at the Fraunhofer IGB by high-performance liquid chromatography (HPLC) as described by Derwenskus et al. [34].

Measured fatty acids, carotenoids, and Chrl within the diet pellets and other parameters are available in [19]. Abbreviations: Suppl, supplementation; ME, metabolizable energy; SFA, saturated fatty acids; UFA, unsaturated fatty acids; PUFA, polyunsaturated fatty acids; MUFA, monounsaturated fatty acids; EPA, eicosapentaenoic acid; n-3:n-6, omega-3-to-omega-6 ratio; Fx, fucoxanthin; Chrl, chrysolaminarin belonging to the β-glycans; PT, microalgae *Phaeodactylum tricornutum*; PT_ Chrl, diet with Chrl-rich PT, PT_EPA/Fx, diet with EPA- and Fx-rich PT. Fatty acids were measured in chow by gas chromatography. Fx was measured by high-performance liquid chromatography.

### 2.2. Mouse Feeding Experiment

A total of 56 6- to 8-week-old female C57BL/6J mice were obtained from the animal care unit of the University of Hohenheim, Germany. The experiment was set up as described by Neumann et al. [19]. All experiments were approved by the local Institutional Animal Care and Use Committee (Regional Council Stuttgart, permit number: V326-15EM). The mice were divided into seven groups, with eight animals each supplemented with a CD diet; a Chrl-rich diet at concentrations of 5, 15, and 25%; and an EPA/Fx diet at concentrations of 5, 15, and 25%. Clinical health scores were assessed daily, and animals were weighed every three days. On days 9 and 14, the animals were housed solitary in metabolic cages (Tecniplast S.p.A, Buguggiate, Italy) to collect 24 h feces and determine food intake. Feces from day 9 were used for energy calculation, and feces from day 14 were used for SCFA measurement and 16-S gut microbiome sequencing. Mice were sacrificed, and organs (spleen, liver, and colon) were removed, weighed, and stored at −80 °C or in 4% PBS-buffered formalin solution (Carl Roth GmbH & Co., Karlsruhe, Germany).

### 2.3. Histological Analyses

Histological analysis was performed as described by Neumann et al. [19]. Formalin-fixed tissue samples of the 15% and 25% dosage diets, as well as the CD, were embedded in paraffin using hematoxylin-eosin (Sigma Aldrich, Schnelldorf, Germany) staining and analyzed. Samples of the ileum and colon were analyzed for cell infiltration (score 0–3) and tissue damage (score 0–3), and livers were scored from 0 to 3 for steatosis, infiltration, and tissue damage. The thickness of the muscularis externa was measured from captured images of the colon and ileum using the scaled ocular (20×) Axio Vision Rel. 4.8 software (magnification 200×, Zeiss, Oberkochen, Germany).

### 2.4. Measurement of Liver Fat Content

The fat content in the liver was measured by Folch extraction but with modifications, as described by Gille et al. [35].

### 2.5. Fatty Acid Analyses in Mouse Tissues and Chow

Liver and fat in white adipose tissue (WAT), especially inguinal (subcutaneous) WAT (iWAT) and epididymal (visceral) WAT (eWAT), were homogenized in 200 µL methanol with a TissueRuptor (Qiagen, Hilden, Germany). Furthermore, fatty acids were analyzed in the chow, the homogenized liver, and fat tissue as described in [36] using an Agilent 7890A (Agilent, Santa Clara, CA, USA) gas chromatograph with a Supelco SBP-PUFA 30 m × 0.32 mm × 0.2 μm column (Sigma-Aldrich, Schnelldorf, Germany) and an FID detector. Results were compared to a certified C4–C24 FAME mix (Supelco-18919-1AMP, Sigma-Aldrich, Schnelldorf, Germany). The analyses covered saturated fatty acids (C 14:0, C16:0, C17:0, C18:0, C20:0, C21:0, C22:0, C23:0, and C24:0) and unsaturated fatty acids (C 14:1, C15:1, C16:1n7, C17:1, 18:1n9c, -t, C18:2n6c, -t, 18:3n6, C18:3n3, C20:1n9, C20:2n6, C20:3n6, C20:5n3, C20:4n6, C22:1n9, C22:2n6, C22:3n6, and C24:1n9). For the measurement of the n-6:n-3 ratio, the totals of all n-6 PUFAs and n-3 PUFAs were calculated and divided by one another.

### 2.6. Real-Time Quantitative Reverse Transcription PCR

RNA was extracted from ileum tissue (50–100 mg) using peqGOLD TriFast (PEQLAB, Erlangen, Germany) according to the manufacturer’s instructions, and 1 µg of RNA was transcribed into cDNA with SuperScript^®^ IV Reverse-Transcriptase (Thermo FisherScientific, Darmstadt, Germany) after DNAse treatment (Promega, Madison, WI, USA). For the real-time PCR, Eva Green Universal PCR master mix (Bio-Rad Laboratories, Munich, Germany) was used to prepare the PCR mix as described in more detail by Zimmermann et al. [37]. The primers are shown in Table 2; the amplification programs for primers were as follows: Tumor necrosis factor-α (TNFα): 95 °C for 3 min, 39 cycles at 95 °C for 5 s, and 60 °C for 10 min; zonula occludens-1 (ZO1), occludin, interleukin (IL)-1β: 95 °C for 3 min, 40 cycles at 95 °C for 5 s, and 60 °C for 10 s. IL-6: 95 °C for 3 min, 45 cycles at 95 °C for 5 s, and 60 °C for 10 s. The comparative CT method was used to determine gene quantity as described in [37].

### 2.7. Fecal Energy Loss and Fecal Energy Ratio

Energy was measured and energy loss was calculated with samples from day 9 as previously described [19]. The fecal energy ratio was calculated by dividing the intake (feed intake in calories) by the ingested energy at day 9.

### 2.8. SCFA Analysis

For SCFA analysis, a minimum of 200 mg of feces was used, following the method described in [7]. However, no dry mass of feces could be determined due to the low amount available. Thus, the SCFA concentrations are given as wet fecal mass. Due to the small masses of feces collected in the cages, some samples could not be measured as the biomass was used as well for microbiota analysis (CD *n* = 5; Chrl5 *n* = 7, Chrl15 *n* = 6, Chrl25 *n* = 3, EPA/Fx5 *n* = 7, EPA/Fx15 *n* = 2, EPA/Fx25 *n* = 6).

### 2.9. Gut Microbiota Analysis

For 16S ribosomal ribonucleic acid (rRNA) gene sequencing, feces samples from day 14 after the start of experimental feeding were used, bacterial DNA was extracted, and 16S Ribosomal RNA (rRNA) gene amplicons of 2 × 300 bp length were sequenced on the MiSeq platform at the University of Minnesota Genomics Center following a protocol similar to that described in [7]. Samples with sequencing reads below 10.000 were excluded, resulting in CD (*n* = 5), Chrl5 (*n* = 8), Chrl15 (*n* = 7), Chrl25 (*n* = 8), EPA/Fx5 (*n* = 4), EPA/Fx15 (*n* = 6), and EPA/Fx25 (*n* = 6), which were included in the microbiome analysis. Microbial alpha and beta diversity and taxonomies from phylum to species were assigned. Before analysis, data were converted into relative abundance, and values below 0.15% were in all samples. This resulted in 151 operational taxonomic units (OTUs).

### 2.10. Statistical Analyses

All parameters were tested for normal distribution using the Kolmogorov–Smirnov test; for normally distributed data, one-way ANOVA was used to compare statistically significant differences (*p* < 0.05) between microalga diet groups and the CD. Variances were tested with the Brown–Forsythe test. Tukey’s multiple comparison post hoc test was used for equal variances, and for unequal variances, Dunnett’s T3 multiple comparisons test was used. Correlation analyses were performed with two-tailed Spearman-rank correlation, with coefficients in the range of 0.0 to 0.3 (0.0 to −0.3) interpreted as a negligible correlation, whereas correlations in the range of 0.3 to 0.5 or −0.3 to −0.5 were regarded as positive or negative correlations, respectively. All statistical analyses were performed using GraphPad Prism version 9.3.1 (GraphPad Software, San Diego, CA, USA).

## 3. Results

### 3.1. Microalgae Diet Acceptance and Effects on Body Weight (Bw) and Energy Uptake

**Figure 1:**
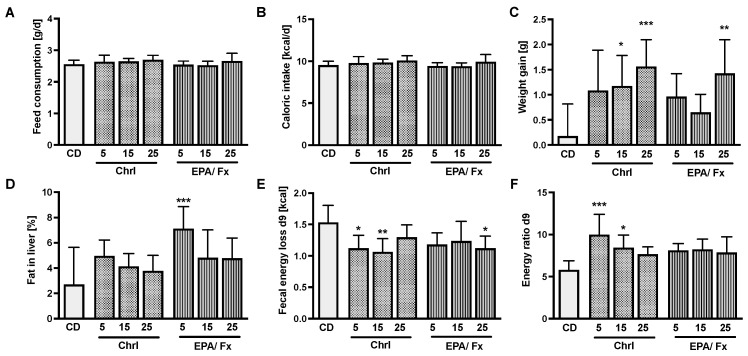
Daily feed (**A**) and caloric intake (**B**) of C57BL/6J mice, body weight gain within 14 days (**C**), and the percentage of liver fat content at day 14 (**D**). The fecal energy loss on day 9 was measured with a calorimeter (**E**), and the energy ratio of day 9 was calculated by dividing the feed intake on day 9 by the fecal energy loss on day 9 (**F**) of C57BL/6J mice. Concentration values are expressed as mean ± SD from *n* = 8 samples per group. Diets supplemented with concentrations of 5%, 15%, and 25%. Statistics: * indicates differences relative to CD. * *p* < 0.05, ** *p* < 0.01, *** *p* < 0.001. Abbreviations: CD, control diet; EPA, eicosapentaenoic acid; Fx, fucoxanthin; Chrl, diet with chrysolaminarin-rich PT; EPA/Fx, diet with EPA- and Fx-rich microalgae PT; d, day.

Feed consumption varied between 2.5 and 2.6 g/day (Figure 1A), and the caloric intake (Figure 1B) was similar in all diets. Both PT diets increased the bw gain (*p* < 0.001), especially in higher doses compared to the PT-free CD (Figure 1C). Only the EPA/Fx5 diet, which did not induce weight gain, increased liver fat compared to the CD (*p* = 0.001; Figure 1D). Fecal energy loss measured on day 9 was lower in the 5 and 15% Chrl-rich diet groups and the 25% EPA/Fx diet group compared to the CD group (*p* = 0.01; Figure 1E). Figure 1F shows a higher energy ratio associated with the Chrl5-rich and −15 diets (*p* = 0.001) compared to the CD. Further analyses, including the weight of organs (not shown) and histological analyses, showed no differences in the CD-fed mice (see Table S1).

### 3.2. Short-Chain Fatty Acids, Markers of Intestinal Permeability, and Inflammation

**Figure 2:**
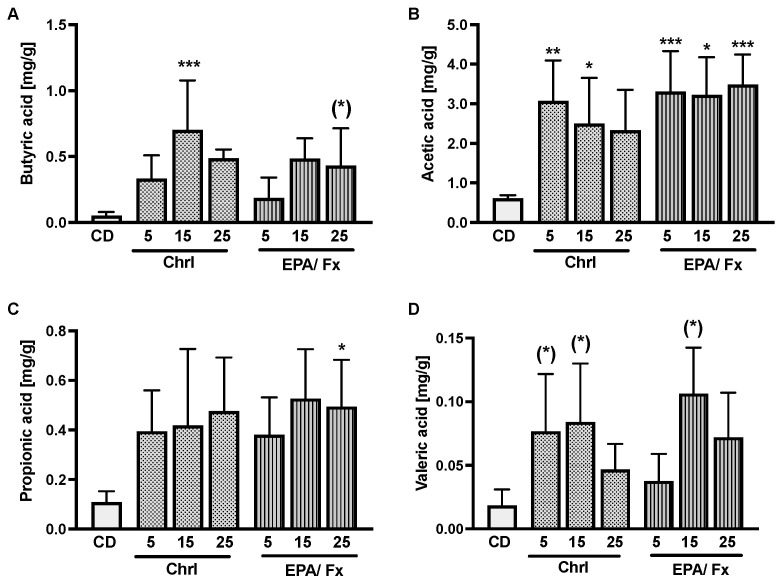
The microalgae diets increased the content of SCFAs, such as butyric acid (**A**), acetic acid (**B**), and propionic acid (**C**), with an increasing tendency of valeric acid (**D**), in feces after supplementation with both versions of the microalgae as measured on day 14 by gas chromatography. Data are expressed as mean ± SD (CD *n* = 5; Chrl5 *n* = 7, Chrl15 *n* = 6, Chrl25 *n* = 3, EPA/Fx5 *n* = 7, EPA/Fx15 *n* = 2, EPA/Fx25 *n* = 6). Statistics: * indicates differences from CD. (*) *p* < 0.1, * *p* < 0.05, ** *p* < 0.01, *** *p* < 0.001. Abbreviations: see Figure 1.

Both microalgae diets caused increased production of SCFA in the feces of mice. The increase was less consistent in butyric acid in association with the Chrl-rich15 diet (*p* < 0.001) and occurred only in trend associated with the EPA/Fx25 (*p* = 0.1) diet compared to the CD (Figure 2A). Acetic acid increased the most in association with PT-rich diets compared to the CD (*p* < 0.001) (Figure 2B). Propionic acid increased only in association with the 25% EPA/Fx diet (*p* = 0.04) (Figure 2C), and valeric acid showed an increasing trend in association with the microalgae diets compared to the CD (*p* = 0.01) (Figure 2D). For intestinal permeability, the relative expressions of zonula occludens protein-1 (ZO1) and occludin were measured in the ileum, and no differences were observed. The proinflammatory tumor necrosis factor-α (TNF-α), interleukin (IL)-6, and IL-1β genes were measured in the ileum. Only TNF-α mRNA expression was higher (*p* < 0.001) in association with the lowest-dose Chrl-rich diet and the two highest-dose Chrl25-rich and EPA/Fx25 diets compared to the CD (Table 3).

### 3.3. Microbiome Analysis in Feces

**Figure 3:**
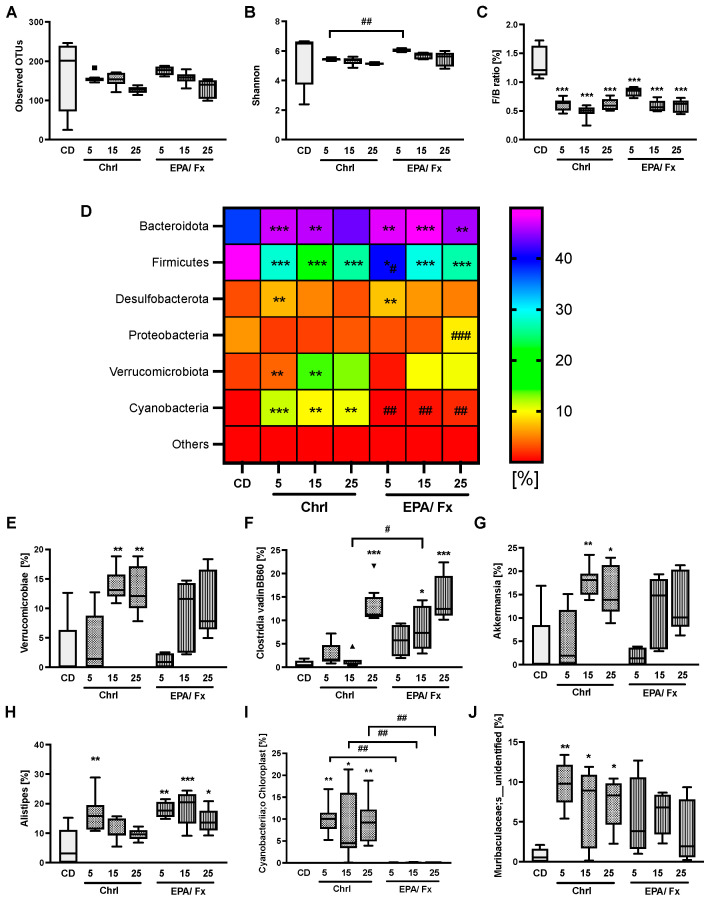
Both PT diets effected changes in the gut microbiome after 14 days of supplementation compared to the CD. The α-diversity, i.e., the species richness (observed OUTs, (**A**)), and the species diversity, i.e., the Shannon index (**B**) were not affected. At the phylum level, the F/B ratio was lower in association with PT diets (**C**), and the relative bacterial abundances [%] of Bacteroidota, Firmicutes, and others changed in association with PT diets compared to the CD (**D**). At the class level, the abundance of *Verrucomicrobiae* was higher in association with Chrl-rich diets (**E**). At the genus level, the abundance of *Clostridia_vadinBB60* (**F**), *Akkermansia* (**G**), and *Alistipes* changed compared to the CD (**H**). The abundances of Cyanobacteriia chloroplast (**I**) and Muribaculaceae at the species level were higher in association with Chrl-rich diets (**J**). Further results are presented in Supplementary Table S2. Data are shown as box plots with median, 25%, and 75% percentiles and a heat map of the phylum as relative bacterial abundances [%] (CD *n* = 5; Chrl5 *n* = 8; Chrl15 *n* = 7; Chrl25 *n* = 8; EPA/Fx5 *n* = 4; EPA/Fx15 *n* = 6; EPA/Fx25 *n* = 6). Statistics: * indicates differences relative to CD, and # indicates the difference between Chrl and EPA/Fx diets. */^#^ *p* < 0.05, **/^##^ *p* < 0.01, ***/^###^ *p* < 0.001. Abbreviations: OTUs, operational taxonomic units; F/B, Firmicutes/Bacteroidota ratio; for other abbreviations, see Table 1 and Figure 1.

Gut microbiome analysis showed no changes in β-diversity as measured by the Bray–Curtis distance and no effect on α-diversity compared to the CD (Figure 3A,B). Taxonomies changed between the microalgae diets and the CD from phylum to species. At the phylum level, Bacteriodota, Firmicutes, Desulfobacterota, Verrucomicrobiota, Cyanobacteria, and Proteobacteria were the most dominant bacteria and changed compared to the CD. The Firmicutes-to-Bacteroidota (F/B) ratio led to a significant reduction in association with all microalgae diets compared to the CD (*p* < 0.001; Figure 3C). In detail, the relative abundance of Bacteroidota was higher in association with all microalgae diets except for the Chrl25-rich diet (*p* < 0.001) (Figure 3D), and a lower abundance was demonstrated for the Firmicutes in association with all microalgae diets compared to the CD (*p* < 0.001). Further changes are shown in Figure 3D and Table S2. At the class level, the abundance of *Verrucomicrobiae* was higher following higher doses of Chrl15-rich and -25 (*p* < 0.001) diets compared to the CD (Figure 3E; other results see Table S2). The order level showed a reduction in *Lachnospirales* after higher doses of both PT biomass supplementations (*p* < 0.01; Table S2). At the genus level, results consistently showed a higher abundance of *Clostridia vadinBB60* in association with both highest-dosed Chrl25 and EPA/Fx15 and 25 diets (*p* < 0.001) compared to CD (Figure 3F). *Akkermansia* abundance was higher in association with both higher-dosed Chrl-rich diets (*p* < 0.001) compared to the CD (Figure 3G). The results were less consistent for *Alistipes*, which presented with higher abundance only in association with the lowest Chrl-rich supplementation (*p* = 0.002) and all EPA/Fx diets (*p* < 0.04) compared to the CD (Figure 3H). At the species level, the abundance of *Cyanobacteriia Chloroplast* was higher in association with all Chrl-rich diets compared to the CD and EPA/Fx diets (*p* < 0.001; Figure 3I). The abundance of *Muribaculaceae*;s_*unidentified* was increased in association with all Chrl-rich (*p* = 0.003; Figure 3J) diets. For more details, see Table S2.

### 3.4. Fatty Acids Measured in the Liver and the White Adipose Tissue (WAT)

**Figure 4:**
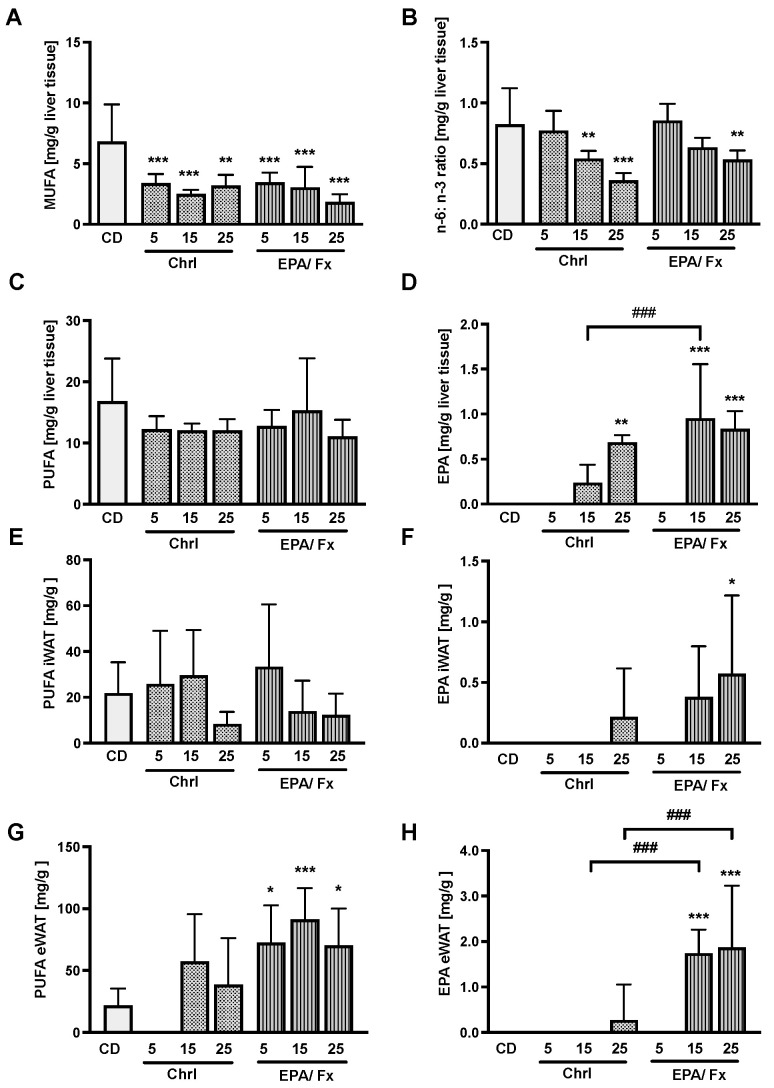
Fatty acids were measured by gas chromatography in the liver (**A**–**D**), subcutaneous WAT (**E**,**F**), and visceral WAT (**G**,**H**). MUFA amounts were lower in association with all microalgae diets (**A**), and the ratio of n-6-to-n-3 PUFA (**B**) decreased in association with higher supplementation of the two microalgae diets. PUFA content was increased in eWAT (**G**) in association with the EPA/Fx diets, and EPA content increased in all tissues with higher supplementation of the EPA/Fx diets and in the liver in association with the Chrl25 diet (**D**,**F**,**H**). Data are expressed as mean ± SD (*n* = 8 per group). Statistics: * indicates difference relative to CD, and ^#^ indicates the difference between Chrl and EPA/Fx diets. * *p* < 0.05, **p < 0.01, ***/^###^ *p* < 0.001. Abbreviations: WAT, white adipose tissue; iWAT, inguinal (subcutaneous) WAT; eWAT; epididymal (visceral) WAT; see Table 1 and Figure 1 for other abbreviations.

Fatty acids were measured in the liver and WAT to compare their bioavailability and differences in absorption levels. Both microalgae diets showed a lower monounsaturated fatty acid (MUFA) concentration (*p* < 0.001) (Figure 4A) and a lower n-6: n-3 ratio in the liver at higher doses compared to the CD (*p* < 0.001; Figure 4B). The PUFA concentration did not show any differences in the liver (Figure 4C) and iWAT (Figure 4E) but higher concentrations in eWAT in association with the EPA/Fx diet compared to the CD (*p* = 0.001; Figure 4G). EPA concentrations increased dose-dependently in the liver (*p* < 0.001; Figure 4D) and fat tissue compared to the CD (Figure 4F,H). Following ingestion of the EPA/Fx diet with higher EPA doses, higher amounts were measured in the liver and eWAT compared to the Chrl diet (Figure 4D,H).

### 3.5. Correlations

**Figure 5:**
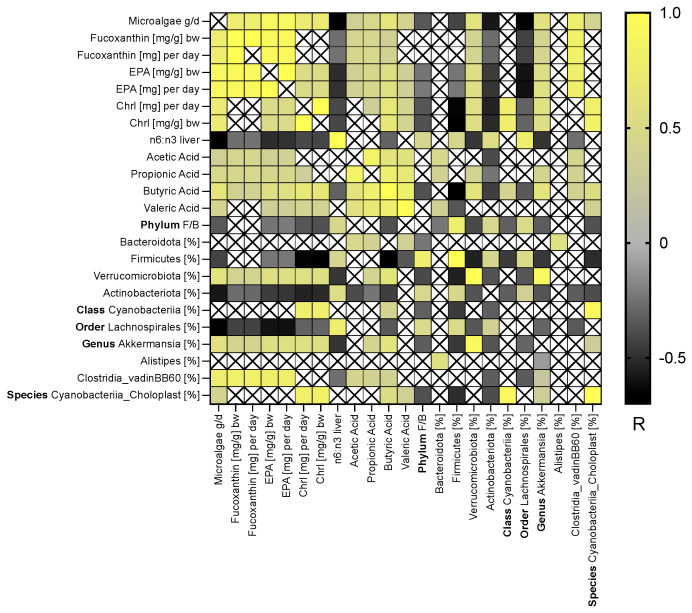
Heat map of Spearman’s rank correlation measured by Spearman rho (R) comparing the microalgae intake/day, EPA, Fx, and Chrl intake mg/day and mg/g bw with the n:6-n:3 ratio in the liver, SCFA amounts (acetic, propionic, butyric, and valeric acid), and gut microbiome as the relative bacterial abundances [%] at the phylum, class, order, genus, and species levels. Abbreviations: X, no significance at *p*-values > 0.05; bw, body weight; EPA, eicosapentaenoic acid; Chrl, chrysolaminarin intake; F/B, Firmicutes/Bacteroidota ratio; for further abbreviations, see Table 1 and Figure 1.

For correlation analysis, the daily Chrl, Fx, and EPA intakes were calculated separately in mg/day and mg/g bw per day and correlated with fecal SCFA levels, the n-6:n-3 ratio in the liver, and the most important bacteria at different levels (Figure 5). The microalgae and EPA intake correlated with all SCFA amounts measured in feces. The daily Fx intake correlated with all SCFA levels except valeric acid, and the Chrl uptake expressed in mg/day correlated with SCFA levels except acetic acid. The Chrl intake expressed in mg/g bw correlated with butyric and valeric acid fecal concentrations. All intakes correlated negatively with the n-6:n-3 ratio in the liver. The intake of the whole microalgae and the components EPA, Fx, and Chrl measured daily was negatively correlated with the F/B ratio, Firmicutes, and Actinobacteria abundance at the phylum level and *Lachnospirales* abundance at the order level. A positive association was observed between the intake and the relative abundance of Verrucomicrobiae at the phylum level and *Akkermansia* at the genus level. *Clostridia vadinBB60* was positively associated with EPA and Fx but not with Chrl intake. Further results showed that a higher abundance of *Cyanobacteriia* at the class level and *Cyanobacteriia_Choroplast* at the genus level is only associated with Chrl intake.

The connection between the SCFA amounts and some bacteria was elucidated. A positive correlation was demonstrated for acetic acid between Bacteriodota at the phylum level and *Clostridia vadinBB60* at the genus level. The Bacteriodota Verrucomicrobiae at the phylum level and *Akkermansia* and *Clostridia vadinBB60* at the genus level are positively correlated with propionic acid. The propionic acid amount and the Actinobacteria abundance are negatively correlated at the phylum level. For butyric acid, a positive correlation was detected between the Verrucomicrobiae at the phylum level; *Cyanobacteriia* at the class level; and *Akkermansia*, *Clostridia vadinBB60*, and *Cyanobacteriia_Choroplast* at the genus level. A negative correlation was shown between the abundance of Firmicutes and Actinobacterota at the phylum level and *Lachnospirales* at the order level. For valeric acid, Bacteroidota and *Cyanobacteriia_Choroplast* were positively correlated at the phylum level, whereas Firmicutes correlated negatively at the phylum level.

## 4. Discussion

In this study, we investigated two biomass samples from the microalgae PT containing different amounts of EPA, Fx, and Chrl due to different cultivation conditions. The investigation in mice comprised acceptance and safety issues, as well as aspects of gut health, such as microbiota and fecal SCFA analyses. Such analyses are also needed to approve microalgae PT biomass as a novel food. This study provides information on the safe intake of microalgae biomass and demonstrates possible beneficial effects on gut health in a preclinical setting.

With respect to safety issues, we assessed the integrity of the intestinal barrier by measuring occludin and zonula occludens 1 in the intestinal mucosa. According to these measurements, the intestinal barrier was not impaired by the PT diets, suggesting the possibility of safe intake of PT. It has been reported that β-glucans can enhance the expression of TJ proteins in obese mice [38] and normal-weight pigs [39]. Although PT contains the β-glucan Chrl, we did not observe a beneficial effect of PT diets on the barrier, likely because our mice were healthy and no severe barrier impairment was expected before the intervention. Other PT ingredients, such as EPA and Fx, might also play a protective role in the recovery of TJ proteins following inflammatory processes [40,41,42]. Whereas no benefit was observed in our healthy mice when fed PT diets, a beneficial effect can be anticipated if the recipients suffer from an impaired gut barrier.

Interestingly, the PT diets did not induce a clear inflammatory response, although we observed an increase in TNF-α mRNA levels in the ileum. However, other proinflammatory cytokine mRNA levels, such as IL-6 and IL-1β, were not affected. A negative correlation was measured between the n-3 PUFA in the liver, which was increased by the PT diets, and pro-inflammatory IL-1β, suggesting an anti-inflammatory effect of PT, which is further supported by the reducing effect of the PT diets on the n-6:n-3 ratio. The Chrl-rich extract of PT (0.06%) has immunomodulatory effects in sea bream [30], which might be related to induction of intestinal TNF-α levels. The increase in TNF-α levels observed in our studies requires further investigation, and measurement inaccuracies must be prevented. According to own previous data, PT intake has anti-inflammatory rather than proinflammatory effects [36].

Considering the medium dose of 15% microalgae content in the diet, we conclude that our data demonstrated a safe intake of Fx of up to 4.3 mg/day in mice with a bw of approx. 18 g (231 mg/kg bw per day). Such doses cannot be easily extrapolated to the human situation. Equivalent amounts in human diets are usually lower because of a slower metabolic turnover in humans compared to rodents; on the other hand, higher doses are often administered for medical use. In a recent pilot trial in humans, we demonstrated a safe intake of up to 30 mg/day of Fx [7], and the EFSA published a recommendation for 15 mg/day of pure Fx extracted from *Undaria pinnatifida thallus* [43]. Further human studies are needed to evaluate safe recommended dietary allowance.

With respect to EPA, this study confirms good bioavailability from both PT diets and a safe intake of EPA of up to 17 mg/day related in mice with a bw of approx. 18 g (920 mg/kg bw per day). As expected, higher EPA concentrations of the EPA/Fx diets resulted in higher absorption of EPA in the liver and fat tissue than the Chrl-rich diets. Regarding the dietary intake limits for Chrl, the present study showed no tissue changes or damage associated with intakes of up to 86.6 mg/day in mice with a bw of approx. 18 g (4621 mg/kg bw). EFSA has already been approved for the human diet with a dose of up to 600 mg/day of -(1,3)/(1,6)-glucan derived from the cell wall of baker’s yeast *Saccharomyces cerevisiae* [44]. A toxicological study in rats demonstrated a safe intake of up to 100 mg/kg bw of this type of β-glucan [45]. Therefore, we assume that the Chrl type of β-(1,3)/(1,6)-glucan derived from PT should also be approved soon. Other microalgae containing β-(1,3)/(1,6)-glucan or β-(1,3)-glucans, such as *Odontalla auritia* and *Euglena gracilis*, are already authorized in the EU [17,46]. A drug derived from *Euglena gracilis* is already on the market for immune stimulation (BioGlenaTM; Algatechnologies Inc. Eilot, Israel). Thus, no safety issues should be expected because of the β-(1,3)/(1,6)-glucan contained in PT; instead, beneficial effects should be assumed, supporting the idea of using PT-based products for human nutrition.

Although a literature search did not reveal any studies reporting infection or intoxication by the diatom PT [47], some recent studies suggest that PT can produce β-N-methylamino-L-alanine (BMAA) under particular culture conditions [48,49]. BMAA is a neurotoxin produced by certain cyanobacteria, diatoms, and dinoflagellates, with adverse effects on humans [50]. BMAA is found in mussels and scallops and is thus transferred to the food chain [51,52]. However, our previous studies in cell culture, in mice, and humans, as well as the present study, did not reveal any signs of neurotoxicity, such as abnormal movement or behavior in the mice, which calls into question the relevance of BMAA in the PT biomass, possibly because of the different culture conditions we used in our studies [7,19,36]. On the other hand, BMAA in PT biomass should be quantified, and the amounts should be toxicologically evaluated in mice and humans to complete a qualified presumption of safety (QPS) procedure used by EFSA Scientific Panels for risk assessment of biological agents [47].

Our preclinical data revealed that PT diets are safe and possibly also healthy, as they showed some beneficial effects on the intestinal microbiota and possibly also on the generation of SCFAs. SCFAs have been attributed to beneficial effects on gut health, nourishing the mucosa, preventing colon cancer, and protecting against leaky gut [53,54]. SCFAs are produced from dietary fibers, such as β−glucans fermented to SCFAs by commensal bacteria [55]. This concept was supported by studies showing an increase in acetic acid and butyric acid after supplementation with β-glucans from barley [56,57], oats [58], and laminarin from *Laminaria* spp. [59,60]. Interestingly, n-3 PUFA has also been found to promote SCFA production. Apart from their anti-inflammatory effects, n-3 PUFAs are considered prebiotics, as studies have provided evidence that n3-PUFAs promote SCFA production [61,62] and an increase in LPS-suppressing bacteria, such as *Bifidobacteria* [63]. The daily intake of Fx may also influence SCFA production, as demonstrated by Sun et al. [32].

Due to the similar structure of Chrl compared to other β-glucans, such as laminarin, and the high content of EPA and Fx, we assumed similar effects. We observed an increase in acetic acid and a higher amount of butyric acid after administration of the Chrl15-rich diet. Correlation analyses confirmed this assumption, and we demonstrated, for the first time, that SCFA levels correlated with Chrl uptake from diet. The current EPA/Fx diet increased acetic acid in association with all three diets. Additionally, correlation analysis confirmed that the highest dose of the diet also increased propionic acid levels. This increase suggests a possible benefit for gut health, as propionic acid is essential for mucosal healing and anti-inflammation [64]. A lower daily dose of 286 mg EPA+DHA did not change SCFA levels in a previous clinical trial conducted by our group after two weeks of PT intake [7]. The results suggest that Chrl, EPA, and Fx could promote SCFA production. Further evaluation is required, as the SCFA changes were minor and not always consistent for the different supplementation groups.

Due to the effects of PT diets on SCFA production in the gut, we also expected changes in the gut microbiome. As shown by other studies, the supplementation of n-3 PUFA, β-glucan [65,66], and Fx [32] increased bacterial diversity. However, in our study, we were not able to confirm these results. However, the PT diets modified the gut microbiome at the phylum level; they reduced the F/B ratio, confirming other studies supplementing with β-glucan from barley [66] and laminarin from macroalgae [67,68]. Correlation analyses revealed that the F/B ratio is negatively associated with microalgae intake, as well as EPA and Chrl intake. Furthermore, the Chrl-rich diets enhanced the abundance of the SCFA-producing *Akkermanisa* sp. [53]. Our data confirm an increase in *Akkermanisa* sp. after supplementation with the β-(1,3)-glucan paramylon, as reported in previous studies [69]. Additionally, n-3 PUFA supplementation is thought to increase *Akkermansia* sp. [70], especially *Akkermansia muciniphila* [71]. Therefore, *Akkermansia* sp. have been identified as “next-generation probiotics” [72]. Our murine data were less clear in this respect. However, a recent human study performed by our group demonstrated that consuming an EPA/Fx-rich PT diet increased *Akkermansia* after 14 days of intervention [7]. We conclude that Chrl-rich PT intake increases the abundance of *Akkermansia*.

The distinctive effect of the Chrl-rich diet could be due to the increase in the abundance of *Cyanobacteriia*_*chloroplasts*, possibly resulting from the formation of chloroplasts following cyanobacterial endosymbiosis [73]. Minor other changes included a lower abundance of *Lachnospirales* associated with PT diets, a butyric acid-producing bacteria [74]. Supplementation with n-3 PUFA was previously to shown to result in an increase in *Lachnospirales* due to conditional promotion by DHA [65]. The fact that the PT diet is mainly rich in EPA could be a possible explanation for the decline. Further changes were observed, including an increase in bacteria involved in mucosal sugar uptake [75], such as species *Muribaculaceae*_*unidentified* associated with the Chrl-rich diet and *Alistepes* in association with the EPA/Fx diet. The higher abundance of *Clostridia_vadinBB60* at the genus level is very poorly classified, and their role in the microbiota [76] requires further investigation.

## 5. Conclusions

Our preclinical study in healthy mice shows that the intake of two types of PT diets containing Chrl, EPA, and Fx as bioactive compounds is safe and well accepted by mice. With the same calorie intake and increased weight gain, we assume energy absorption. Due to the increase in TNF-α levels, further investigation is needed, and the mean dose of the two PT diets is described as the safest diet. Health benefits, such as the increase in SCFA, need to be further investigated, as the available results are inconsistent. The microbiota results could show positive effects, such as a decrease in the F/B ratio, and the Chrl-rich diet mainly led to an increase in the SCFA-producing bacteria *Akkermansia* sp. The data suggest that PT diets could be suitable for human nutrition, which must be confirmed in human trials.

## Figures and Tables

**Table 1:** Food composition of different diets.

Treatment	Suppl	ME	SFA	UFA	PUFA	MUFA	EPA	n-3:n-6	Fx	β-Carotin	Chrl
	[%]	[MJ/kg]	[g/kg]	[g/kg]	[g/kg]	[g/kg]	[g/kg]		[g/kg]	[g/kg]	[g/kg]
Control diet		15.6	9.33	11.18	3.60	7.58	0.00	0.08	0	0	0
PT_Chrl	5	15.6	16.98	30.83	51.45	21.59	0.00	0.10	0.14	0.02	10.92
	15	15.6	19.24	43.19	45.57	24.68	5.11	0.31	0.42	0.05	32.77
	25	15.6	19.11	49.58	27.50	24.41	8.32	0.90	0.71	0.09	54.62
PT_EPA/Fx	5	15.6	20.49	33.81	68.14	24.534	3.32	0.16	0.57	0.07	0.66
	15	15.6	22.44	52.96	72.56	26.68	11.14	0.30	1.71	0.22	1.98
	25	15.6	21.23	53.57	60.74	24.01	15.17	0.47	2.85	0.37	3.3

**Table 2:** Primer sequences used in quantitative real-time PCR.

Primer	Forward (5′-3′)	Reverse (5′-3′)
Occludin	ACTCCTCCAATGGACAAGTG	CCCCACCTGTCGTGTAGTCT
ZO1	CCACCTCTGTCCAGCTCTTC	CACCGGAGTGATGGTTTTCT
TNFα	ACCACCATCAAGGACTCA	AGGTCTGAAGGTAGGAAG
IL-1β	ACGGATTCCATGGTGAAGTC	GAGTGTGGATCCCAAGCAAT
IL-6	AGTCACAGAAGGAGTGGCTA	CTGACCACAGTGAGGAATGT

Abbreviations: ZO1, zonula occludens-1; TNFα, tumor necrosis factor-α; IL-1β, inflammatory-1β; IL-6, inflammatory-6.

**Table 3:** Intestinal and inflammatory markers measured in the ileum tissue.

Treatment	Suppl [%]	ZO1	Occludin	TNF-α	IL-6	IL-1β
CD		29.94 ± 15	39.17 ± 27	10.06 ± 8.6	49.75 ± 82	5.850 ± 2.8
Chrl	5	59.92 ± 30	46.56 ± 24	69.87 ± 28 **	148.8 ± 127	25.23 ± 15
	15	25.67 ± 21	28.50 ± 18	22.58 ± 17	113.2 ± 135	14.18 ± 14
	25	25.31 ± 26	48.57 ± 40	118.5 ± 94 **	8.526 ± 8	29.60 ± 49
EPA/Fx	5	24.15 ± 22	39.07 ± 26	32.88 ± 26	244.8± 344	18.73 ± 14
	15	16.90 ± 8	27.18± 15	27.57± 18	93.87 ± 129	7.355 ± 5.3
	25	96.19 ± 161	75.82 ± 105	62.58± 40 *	152.3 ± 99	32.19 ± 48

Increase in TNF-α associated with Chrl5/25 and EPA/Fx25 diets compared to CD analyzed by ANOVA. Diets supplemented with 5%, 15%, and 25%. Statistics: * indicates differences relative to CD. * *p* < 0.05, ** *p* < 0.01. Abbreviations: CD, control diet; Suppl, supplementation; ZO1, zonula occludens-1; TNF-α, tumor necrosis factor-α; IL-6, inflammatory-6; IL-1β, inflammatory-1β; for other abbreviations, see Table 1 and Figure 1.

## Data Availability

Data are available upon justified request to the corresponding author.

## Supplementary Materials

The following supporting information can be downloaded at: https://www.mdpi.com/article/10.3390/nu14122504/s1, Table S1: Histological scores of the liver, ileum, and colon after 14 days of diet supplementation.; Table S2: Bacterial taxa in feces after 14 days supplementation of the CD and Chrl-rich and EPA/Fx PT diets.

## References

[1] Grosso G., Mateo A., Rangelov N., Buzeti T., Birt C., on behalf of the Food and Nutrition Section of the European Public Health Association (2020). Nutrition in the Context of the Sustainable Development Goals. Eur. J. Public Health.

[2] Barkia I., Saari N., Manning S.R. (2019). Microalgae for High-Value Products Towards Human Health and Nutrition. Mar. Drugs.

[3] Yang R., Wei D., Xie J. (2020). Diatoms as Cell Factories for High-Value Products: Chrysolaminarin, Eicosapentaenoic Acid, and Fucoxanthin. Crit. Rev. Biotechnol..

[4] Bertrand M. (2010). Carotenoid Biosynthesis in Diatoms. Photosynth. Res..

[5] Mikami K., Hosokawa M. (2013). Biosynthetic Pathway and Health Benefits of Fucoxanthin, an Algae-Specific Xanthophyll in Brown Seaweeds. Int. J. Mol. Sci..

[6] Gille A., Neumann U., Louis S., Bischoff S.C., Briviba K. (2018). Microalgae as a Potential Source of Carotenoids: Comparative Results of an in Vitro Digestion Method and a Feeding Experiment with C57BL/6J Mice. J. Funct. Foods.

[7] Stiefvatter L., Lehnert K., Frick K., Montoya-Arroyo A., Frank J., Vetter W., Schmid-Staiger U., Bischoff S.C. (2021). Oral Bioavailability of Omega-3 Fatty Acids and Carotenoids from the Microalgae Phaeodactylum Tricornutum in Healthy Young Adults. Mar. Drugs.

[8] Bae M., Kim M.-B., Park Y.-K., Lee J.-Y. (2020). Health Benefits of Fucoxanthin in the Prevention of Chronic Diseases. Biochim. Biophys. Acta BBA Mol. Cell Biol. Lipids.

[9] Kim J.H., Kim S.M., Cha K.H., Mok I.-K., Koo S.Y., Pan C.-H., Lee J.K. (2016). Evaluation of the Anti-Obesity Effect of the Microalga Phaeodactylum Tricornutum. Appl. Biol. Chem..

[10] Peng J., Yuan J.-P., Wu C.-F., Wang J.-H. (2011). Fucoxanthin, a Marine Carotenoid Present in Brown Seaweeds and Diatoms: Metabolism and Bioactivities Relevant to Human Health. Mar. Drugs.

[11] Gao B., Chen A., Zhang W., Li A., Zhang C. (2017). Co-Production of Lipids, Eicosapentaenoic Acid, Fucoxanthin, and Chrysolaminarin by Phaeodactylum Tricornutum Cultured in a Flat-Plate Photobioreactor under Varying Nitrogen Conditions. J. Ocean Univ. China.

[12] Becker W. (2004). 18 Microalgae in Human and Animal Nutrition. Handbook of Microalgal Culture: Biotechnology and Applied Phycology.

[13] Burdge G.C. (2006). Metabolism of α-Linolenic Acid in Humans. Prostaglandins Leukot. Essent. Fatty Acids.

[14] Bresson J.L., Fairweather-Tait S., Flynn A., Golly I., Korhonen H., Lagiou P., Løvik M., Marchelli R., Martin A., Moseley B. (2010). Scientific Opinion on Dietary Reference Values for Fats, Including Saturated Fatty Acids, Polyunsaturated Fatty Acids, Monounsaturated Fatty Acids, Trans Fatty Acids, and Cholesterol. EFSA J..

[15] Serhan C.N. (2014). Novel Pro-Resolving Lipid Mediators in Inflammation Are Leads for Resolution Physiology. Nature.

[16] Coll M., Libralato S., Tudela S., Palomera I., Pranovi F. (2008). Ecosystem Overfishing in the Ocean. PLoS ONE.

[17] European Commission EUR-Lex—32017R2470—Durchführungsverordnung (EU) 2017/2470 (2017). https://eur-lex.europa.eu/eli/reg_impl/2017/2470/oj/deu.

[18] Xia S., Gao B., Fu J., Xiong J., Zhang C. (2018). Production of Fucoxanthin, Chrysolaminarin, and Eicosapentaenoic Acid by Odontella Aurita under Different Nitrogen Supply Regimes. J. Biosci. Bioeng..

[19] Neumann U., Derwenskus F., Gille A., Louis S., Schmid-Staiger U., Briviba K., Bischoff S. (2018). Bioavailability and Safety of Nutrients from the Microalgae Chlorella Vulgaris, Nannochloropsis Oceanica and Phaeodactylum Tricornutum in C57BL/6 Mice. Nutrients.

[20] Caballero M.A., Jallet D., Shi L., Rithner C., Zhang Y., Peers G. (2016). Quantification of Chrysolaminarin from the Model Diatom Phaeodactylum Tricornutum. Algal Res..

[21] Beattie A., Hirst E.L., Percival E. (1961). Studies on the Metabolism of the Chrysophyceae. Comparative Structural Investigations on Leucosin (Chrysolaminarin) Separated from Diatoms and Laminarin from the Brown Algae. Biochem. J..

[22] Yin G., Li W., Lin Q., Lin X., Lin J., Zhu Q., Jiang H., Huang Z. (2014). Dietary Administration of Laminarin Improves the Growth Performance and Immune Responses in Epinephelus Coioides. Fish Shellfish Immunol..

[23] Ciecierska A., Drywień M.E., Hamulka J., Sadkowski T. (2019). Nutraceutical Functions of Beta-Glucans in Human Nutrition. Rocz. Państw. Zakładu Hig..

[24] Zhu F., Du B., Xu B. (2016). A Critical Review on Production and Industrial Applications of Beta-Glucans. Food Hydrocoll..

[25] Jayachandran M., Chen J., Chung S.S.M., Xu B. (2018). A Critical Review on the Impacts of β-Glucans on Gut Microbiota and Human Health. J. Nutr. Biochem..

[26] Kadam S.U., Tiwari B.K., O’Donnell C.P. (2015). Extraction, Structure and Biofunctional Activities of Laminarin from Brown Algae. Int. J. Food Sci. Technol..

[27] Kusaikin M.I., Ermakova S.P., Shevchenko N.M., Isakov V.V., Gorshkov A.G., Vereshchagin A.L., Grachev M.A., Zvyagintseva T.N. (2010). Structural Characteristics and Antitumor Activity of a New Chrysolaminaran from the Diatom Alga Synedra Acus. Chem. Nat. Compd..

[28] Xia S., Gao B., Li A., Xiong J., Ao Z., Zhang C. (2014). Preliminary Characterization, Antioxidant Properties and Production of Chrysolaminarin from Marine Diatom Odontella Aurita. Mar. Drugs.

[29] Carballo C., Chronopoulou E.G., Letsiou S., Maya C., Labrou N.E., Infante C., Power D.M., Manchado M. (2018). Antioxidant Capacity and Immunomodulatory Effects of a Chrysolaminarin-Enriched Extract in Senegalese Sole. Fish Shellfish Immunol..

[30] Reis B., Gonçalves A.T., Santos P., Sardinha M., Conceição L.E.C., Serradeiro R., Pérez J., Calduch J., Schmid U., Frick K. (2021). Immune Status and Hepatic Antioxidant Capacity of Gilthead Seabream Sparus Aurata Juveniles Fed Yeast and Microalga Derived Β-Glucans. Mar. Drugs.

[31] Vijay A., Astbury S., Le Roy C., Spector T.D., Valdes A.M. (2021). The Prebiotic Effects of Omega-3 Fatty Acid Supplementation: A Six-Week Randomised Intervention Trial. Gut Microbes.

[32] Sun X., Zhao H., Liu Z., Sun X., Zhang D., Wang S., Xu Y., Zhang G., Wang D. (2020). Modulation of Gut Microbiota by Fucoxanthin During Alleviation of Obesity in High-Fat Diet-Fed Mice. J. Agric. Food Chem..

[33] Atanasov J., Schlörmann W., Trautvetter U., Glei M. (2020). The Effects of β-Glucans on Intestinal Health. Ernahrungs Umsch..

[34] Derwenskus F., Metz F., Gille A., Schmid-Staiger U., Briviba K., Schließmann U., Hirth T. (2019). Pressurized Extraction of Unsaturated Fatty Acids and Carotenoids from Wet Chlorella Vulgaris and Phaeodactylum Tricornutum Biomass Using Subcritical Liquids. GCB Bioenergy.

[35] Gille A., Stojnic B., Derwenskus F., Trautmann A., Schmid-Staiger U., Posten C., Briviba K., Palou A., Bonet M.L., Ribot J. (2019). A Lipophilic Fucoxanthin-Rich Phaeodactylum Tricornutum Extract Ameliorates Effects of Diet-Induced Obesity in C57BL/6J Mice. Nutrients.

[36] Neumann U., Louis S., Gille A., Derwenskus F., Schmid-Staiger U., Briviba K., Bischoff S.C. (2018). Anti-Inflammatory Effects of Phaeodactylum Tricornutum Extracts on Human Blood Mononuclear Cells and Murine Macrophages. J. Appl. Phycol..

[37] Zimmermann J., De Fazio L., Kaden-Volynets V., Hitzmann B., Bischoff S.C. (2022). Consumption of Yeast-Fermented Wheat and Rye Breads Increases Colitis and Mortality in a Mouse Model of Colitis. Dig. Dis. Sci..

[38] Drew J.E., Reichardt N., Williams L.M., Mayer C.-D., Walker A.W., Farquharson A.J., Kastora S., Farquharson F., Milligan G., Morrison D.J. (2018). Dietary Fibers Inhibit Obesity in Mice, but Host Responses in the Cecum and Liver Appear Unrelated to Fiber-Specific Changes in Cecal Bacterial Taxonomic Composition. Sci. Rep..

[39] Wu X., Chen D., Yu B., Luo Y., Zheng P., Mao X., Yu J., He J. (2018). Effect of Different Dietary Non-Starch Fiber Fractions on Growth Performance, Nutrient Digestibility, and Intestinal Development in Weaned Pigs. Nutrition.

[40] Hwang P.-A., Phan N.N., Lu W.-J., Ngoc Hieu B.T., Lin Y.-C. (2016). Low-Molecular-Weight Fucoidan and High-Stability Fucoxanthin from Brown Seaweed Exert Prebiotics and Anti-Inflammatory Activities in Caco-2 Cells. Food Nutr. Res..

[41] Xiao K., Liu C., Qin Q., Zhang Y., Wang X., Zhang J., Odle J., Lin X., Hu C.-A.A., Liu Y. (2020). EPA and DHA Attenuate Deoxynivalenol-Induced Intestinal Porcine Epithelial Cell Injury and Protect Barrier Function Integrity by Inhibiting Necroptosis Signaling Pathway. FASEB J. Off. Publ. Fed. Am. Soc. Exp. Biol..

[42] Li Q., Zhang Q., Wang M., Zhao S., Xu G., Li J. (2008). N-3 Polyunsaturated Fatty Acids Prevent Disruption of Epithelial Barrier Function Induced by Proinflammatory Cytokines. Mol. Immunol..

[43] EFSA (2009). Scientific Opinion on the Substantiation of Health Claims Related to Undaria Pinnatifida (Harvey) Suringar and Maintenance or Achievement of a Normal Body Weight (ID 2345) Pursuant to Article 13(1) of Regulation (EC) No 1924/2006. EFSA J..

[44] EFSA (2011). Scientific Opinion on the Safety of ‘Yeast Beta-Glucans’ as a Novel Food Ingredient. EFSA J..

[45] Babíček K., Čechová I., Simon R.R., Harwood M., Cox D.J. (2007). Toxicological Assessment of a Particulate Yeast (1,3/1,6)-β-d-Glucan in Rats. Food Chem. Toxicol..

[46] Commission Implementing Regulation (EU) 2020/1820 of 2 December 2020 Authorising the Placing on the Market of Dried Euglena Gracilis as a Novel Food. https://eur-lex.europa.eu/legal-content/EN/TXT/HTML/?uri=CELEX:32020R1820.

[47] Hazards (BIOHAZ) E.P. (2019). on B.; Koutsoumanis, K.; Allende, A.; Alvarez-Ordóñez, A.; Bolton, D.; Bover-Cid, S.; Chemaly, M.; Davies, R.; De Cesare, A.; Hilbert, F.; et al. Update of the List of QPS-Recommended Biological Agents Intentionally Added to Food or Feed as Notified to EFSA 10: Suitability of Taxonomic Units Notified to EFSA until March 2019. EFSA J..

[48] Lage S., Ström L., Godhe A., Rydberg S. (2019). Kinetics of β-N-Methylamino-L-Alanine (BMAA) and 2, 4-Diaminobutyric Acid (DAB) Production by Diatoms: The Effect of Nitrogen. Eur. J. Phycol..

[49] Réveillon D., Séchet V., Hess P., Amzil Z. (2016). Production of BMAA and DAB by Diatoms (Phaeodactylum Tricornutum, Chaetoceros Sp., Chaetoceros Calcitrans and, Thalassiosira Pseudonana) and Bacteria Isolated from a Diatom Culture. Harmful Algae.

[50] Van Onselen R., Downing T.G. (2019). β-N-Methylamino-L-Alanine Inhibits Human Catalase Activity: Possible Implications for Neurodegenerative Disease Development. Int. J. Toxicol..

[51] Salomonsson M.L., Fredriksson E., Alfjorden A., Hedeland M., Bondesson U. (2015). Seafood Sold in Sweden Contains BMAA: A Study of Free and Total Concentrations with UHPLC–MS/MS and Dansyl Chloride Derivatization. Toxicol. Rep..

[52] Lance E., Arnich N., Maignien T., Biré R. (2018). Occurrence of β-N-Methylamino-l-Alanine (BMAA) and Isomers in Aquatic Environments and Aquatic Food Sources for Humans. Toxins.

[53] Parada Venegas D., De la Fuente M.K., Landskron G., González M.J., Quera R., Dijkstra G., Harmsen H.J.M., Faber K.N., Hermoso M.A. (2019). Short Chain Fatty Acids (SCFAs)-Mediated Gut Epithelial and Immune Regulation and Its Relevance for Inflammatory Bowel Diseases. Front. Immunol..

[54] Sivaprakasam S., Prasad P.D., Singh N. (2016). Benefits of Short-Chain Fatty Acids and Their Receptors in Inflammation and Carcinogenesis. Pharmacol. Ther..

[55] Bischoff S.C. (2011). “Gut Health”: A New Objective in Medicine?. BMC Med..

[56] Chen H., Nie Q., Xie M., Yao H., Zhang K., Yin J., Nie S. (2019). Protective Effects of β-Glucan Isolated from Highland Barley on Ethanol-Induced Gastric Damage in Rats and Its Benefits to Mice Gut Conditions. Food Res. Int..

[57] Belobrajdic D.P., Jobling S.A., Morell M.K., Taketa S., Bird A.R. (2015). Wholegrain Barley β-Glucan Fermentation Does Not Improve Glucose Tolerance in Rats Fed a High-Fat Diet. Nutr. Res..

[58] Qu X., Nazarenko Y., Yang W., Nie Y., Zhang Y., Li B. (2021). Effect of Oat β-Glucan on the Rheological Characteristics and Microstructure of Set-Type Yogurt. Molecules.

[59] Rattigan R., Sweeney T., Maher S., Thornton K., Rajauria G., O’Doherty J. (2019). Laminarin-Rich Extract Improves Growth Performance, Small Intestinal Morphology, Gene Expression of Nutrient Transporters and the Large Intestinal Microbial Composition of Piglets during the Critical Post-Weaning Period. Br. J. Nutr..

[60] Rattigan R., O’Doherty J.V., Vigors S., Ryan M.T., Sebastiano R.S., Callanan J.J., Thornton K., Rajauria G., Margassery L.M., Dobson A.D.W. (2020). The Effects of the Marine-Derived Polysaccharides Laminarin and Chitosan on Aspects of Colonic Health in Pigs Challenged with Dextran Sodium Sulphate. Mar. Drugs.

[61] Watson H., Mitra S., Croden F.C., Taylor M., Wood H.M., Perry S.L., Spencer J.A., Quirke P., Toogood G.J., Lawton C.L. (2018). A Randomised Trial of the Effect of Omega-3 Polyunsaturated Fatty Acid Supplements on the Human Intestinal Microbiota. Gut.

[62] Zhu L., Sha L., Li K., Wang Z., Wang T., Li Y., Liu P., Dong X., Dong Y., Zhang X. (2020). Dietary Flaxseed Oil Rich in Omega-3 Suppresses Severity of Type 2 Diabetes Mellitus via Anti-Inflammation and Modulating Gut Microbiota in Rats. Lipids Health Dis..

[63] Costantini L., Molinari R., Farinon B., Merendino N. (2017). Impact of Omega-3 Fatty Acids on the Gut Microbiota. Int. J. Mol. Sci..

[64] Smith P.M., Howitt M.R., Panikov N., Michaud M., Gallini C.A., Bohlooly Y.M., Glickman J.N., Garrett W.S. (2013). The Microbial Metabolites, Short-Chain Fatty Acids, Regulate Colonic Treg Cell Homeostasis. Science.

[65] Menni C., Zierer J., Pallister T., Jackson M.A., Long T., Mohney R.P., Steves C.J., Spector T.D., Valdes A.M. (2017). Omega-3 Fatty Acids Correlate with Gut Microbiome Diversity and Production of N-Carbamylglutamate in Middle Aged and Elderly Women. Sci. Rep..

[66] Wang Y., Ames N.P., Tun H.M., Tosh S.M., Jones P.J., Khafipour E. (2016). High Molecular Weight Barley β-Glucan Alters Gut Microbiota Toward Reduced Cardiovascular Disease Risk. Front. Microbiol..

[67] Cui Y., Zhu L., Li Y., Jiang S., Sun Q., Xie E., Chen H., Zhao Z., Qiao W., Xu J. (2021). Structure of a Laminarin-Type β-(1→3)-Glucan from Brown Algae Sargassum Henslowianum and Its Potential on Regulating Gut Microbiota. Carbohydr. Polym..

[68] Nguyen S.G., Kim J., Guevarra R.B., Lee J.-H., Kim E., Kim S., Unno T. (2016). Laminarin Favorably Modulates Gut Microbiota in Mice Fed a High-Fat Diet. Food Funct..

[69] Taylor H.B., Gudi R., Brown R., Vasu C. (2020). Dynamics of Structural and Functional Changes in Gut Microbiota during Treatment with a Microalgal β-Glucan, Paramylon and the Impact on Gut Inflammation. Nutrients.

[70] Aparicio E., Martín-Grau C., Bedmar C., Serrat Orus N., Basora J., Arija V., The ECLIPSES Study Group (2021). Maternal Factors Associated with Levels of Fatty Acids, Specifically n-3 PUFA during Pregnancy: ECLIPSES Study. Nutrients.

[71] Caesar R., Tremaroli V., Kovatcheva-Datchary P., Cani P.D., Bäckhed F. (2015). Crosstalk between Gut Microbiota and Dietary Lipids Aggravates WAT Inflammation through TLR Signaling. Cell Metab..

[72] Cani P.D., Van Hul M. (2015). Novel Opportunities for Next-Generation Probiotics Targeting Metabolic Syndrome. Curr. Opin. Biotechnol..

[73] Sato N. (2021). Are Cyanobacteria an Ancestor of Chloroplasts or Just One of the Gene Donors for Plants and Algae?. Genes.

[74] Louis P., Flint H.J. (2017). Formation of Propionate and Butyrate by the Human Colonic Microbiota. Environ. Microbiol..

[75] Pereira F.C., Wasmund K., Cobankovic I., Jehmlich N., Herbold C.W., Lee K.S., Sziranyi B., Vesely C., Decker T., Stocker R. (2020). Rational Design of a Microbial Consortium of Mucosal Sugar Utilizers Reduces Clostridiodes Difficile Colonization. Nat. Commun..

[76] Richards P., Fothergill J., Bernardeau M., Wigley P. (2019). Development of the Caecal Microbiota in Three Broiler Breeds. Front. Vet. Sci..

