# Supplementary material for: The Microalgae Phaeodactylum tricornutum Is Well Suited as a Food with Positive Effects on the Intestinal Microbiota and the Generation of SCFA: Results from a Pre-Clinical Study
Source: Nutrients. 2022 Jun 16;14(12):2504. doi: 10.3390/nu14122504 (PMC9229211; doi:10.3390/nu14122504)
Supplement: Supplementary file 1 [file nutrients-14-02504-s001.zip › nutrients-1764839-supplementary.pdf]

Supplementary Materials

Table S1 Histological scores of the liver, ileum, and colon after 14 days of diet supplementation.

Diet	Supplementation [%]	Liver Score	Cell Damage Ileum	Infil Ileum	Cell Damage Colon	Infil Colon
**CD**		± 0.1	0.0 ± 0.0	0.1 ± 0.1	0.0 ± 0.0	0.0 ± 0.1
**Chrl**	15	0.2 ± 0.4	0.0 ± 0.0	0.3 ± 0.9	0.0 ± 0.0	0.2 ± 0.4
	25	0.3 ± 0.6	0.1 ± 0.3	0.1 ± 0.3	0.0 ± 0.0	0.2± 0.4
**EPA/ Fx**	15	0.2 ± 0.4	0.0 ± 0.0	0.1 ± 0.3	0.2 ± 0.4	0.1 ± 0.3
	25	0.4 ± 0.5	0.0 ± 0.0	0.0 ± 0.1	0.0 ± 0.0	0.0 ± 0.1

The ANOVA showed no significant differences. Histological scores were measured after the diets were supplemented to 15% and 25%. Data are expressed as mean ± SD (n= 4). Abbreviations: Infil, Infiltration; other abbreviations see Table1.

Table S2. Bacterial taxa in feces after 14 days supplementation of the CD and Chrl- rich and EPA/Fx PT diets.

Treatments	CD	Chrl5	Chrl15	Chrl25	EPA/Fx5	EPA/Fx15	EPA/Fx25
**Phylum**							
**Bacteroidota (B)**	37.4±3.4	46.8±4.0***	46.2±2.6**	43.6±3.1	47.5±2.2**	50.6±4.1***	45.6±4.7**
**Firmicutes (F)**	49.7±8.0	28.2±2.9***	21.9±26.3***	26.3±3.3***	39.4±2.1*	29.4±4.3***	26.8±4.7***
**F/B ratio**	1.3±0.3	0.6±0.1***	0.48±0.1***	0.60±0.1***	0.8±0.1***	0.6±0.01***	0.6±0.1***
**Verrucomicrobiota**	2.5±5.6	3.9±5.0	14.02±2.7**	13.08± 4.0**	1.094±1.3	9.0±6.1	10.3±5.4
**Actinobacteriota**	1.0±1.5	0.05±0.0*	0.00±0.0*	0.0±0.0*	0.06±0.0	0.04±0.0*	0.00±0.0*
**Desulfobacterota**	3.1±2.3	7.1±2.3**	5.1±1.6	3.2±1.1	7.6±0.8**	5.9±1.3	5.0±1.0
**Cyanobacteria**	0.3±0.3	11.4±4.0***	9.8±7.7**	10.4±5.4**	0.4±0.3##	1.0±0.3#	1.8±0.9#
**Class**							
**Bacteroidia**	37.4±3.2	46.8±4.0***	46.2±2.6**	43.6±3.1	47.5±2.3**	50.6±4.1***	45.6±4.7**
**Clostridia**	31.4±17.9	26.4±3.2	20.7±5.1	24.8±3.5	37.8±2.1	28.4±4.3	24.6±3.8
**Verrucomicrobiae**	2.6±5.7	3.9±5.0	14.0±2.7**	13.1±3.9**	1.1±1.3	9.0±6.1	10.3±5.5
**Cyanobacteriia**	0.02±0.05	11.1±4.0**	8.8±7.8	9.5±5.0*	0.08±0.06##	0.09±0.03	0.1±0.1#
**Order**							
**Lachnospirales**	20.0±11.7	13.5±3.4	8.2±4.6**	6.1±2.7***	16.4±4.1	7.9±0.4*	5.1±3.0***
**Oscillospirales**	9.5±5.4	9.7±1.8	9.8±1.9	6.3±1.3	18.3±2.0**###	13.4±1.7	7.3±4.8
**Genus**	CD	Chrl5	Chrl15	Chrl25	EPA/Fx5	EPA/Fx15	EPA/Fx25
**Muribaculaceae**	17.1±14.1	27.4±3.9	28.1±5.6	26.8±4.0	31.8±3.5*	25.4±3.3	16.7±3.2

**Alistipes**	5.1±6.4	16.5±6.2**	10.7±3.6	9.6±1.8	17.9±2.7**	18.7±5.9***	14.3±4.0*
**Parabacteroides**	14.8±17.4	4.8±3.5	7.7±8.4	8.6±3.8	8.9±8.4	10.7±1.1	14.8±3.9
**Dubosiella**	8.4±8.0	1.5±1.9	0.6±0.5	1.0±0.7	1.4±1.2	0.5±0.2	0.8±0.7
**Clostridia_vadinBB60_group**	0.6±0.8	2.8±2.3	1.6±1.3	13.0±3.9***	5.7±3.5	8.3±4.7*#	14.6±4.8***
**Parasutterella**	6.8±5.9	2.7±1.4	2.1±0.7*	3.2±1.2	3.8±1.7	2.7±1.1	5.8±1.5
**Akkermansia**	3.4±7.6	5.1±6.2	17.8±3.3**	15.4±5.2*	1.7±1.9	11.6±7.7	12.7±6.3
**Bacteroides**	3.7±5.6	5.9±3.7	5.4±7.1	1.4±1.2	3.5±2.1	1.0±0.1	1.3±1.3

**Species, OTUs**	CD	Chrl5	Chrl15	Chrl25	EPA/Fx5	EPA/Fx15	EPA/Fx25
**OTU17: Muribaculaceae;s__uncultured_bacterium**	4.0±3.2	3.6±1.4	5.7±1.9	4.3±0.8	6.8±3.8	8.3±2.1*	5.3±2.1
**OTU18: Muribaculaceae;s__ unidentified**	0.8±0.9	10.0±2.7**	7.1±4.6*	7.4±2.9*	5.3±5.1	6.1±2.6	3.6±3.8
**OTU 22: Alistipes;__unidentified**	3.7±4.6	11.5±4.4**	8.47±2.8	8.2±1.7	11.8±2.0*	13.7±4.8***	10.9±3.6*
**OTU 24: Parabacteroides_goldsteinii**	12.9±16.3	3.9±2.7	6.0±6.0	7.5±3.5	5.8±5.5	9.6±3.4	11.3±3.2
**OTU 32: Cyanobacteriia;o__Chloroplast;f__Chloroplast;g__Chloroplast;__unidetified**	0.0±0.0	10.2±3.6**	8.8±7.8*	9.5±5.0**	0.1±0.06##	0.1±0.06##	0.1±0.1##
**OTU 35: Desulfovibrionaceae;g__uncultured;s__uncultured_bacterium**	3.0±2.4	7.0±2.4**	4.6±1.6	2.9±1.1	7.3±1.1**	5.0±1.1	4.7±1.1
**OTU 54: Clostridia_vadinBB60_group;__unidentified**	0.16±	0.8±1.1	0.4±0.3	8.8±1.3***	0.4±0.6	2.3±1.2	4.1±2.3**###
**OTU 145: Burkholderiales_bacterium**	5.7±6.1	2.0±1.0	1.7±0.5*	2.7±0.9	2.5±1.0	2.1±0.7	4.6±1.2

**OTU150: Akkermansia;__unidentified**	2.5±5.7	4.1±5.3	14.0±2.7**	13.1±3.9**	1.1±1.3	10.0±6.0	10.4±5.4

Values are expressed as the relative bacterial abundances [%] (CD n = 5; Chrl5 n = 8; Chrl15 n = 7; Chrl25 n = 8; EPA/Fx5 n = 4; EPA/Fx15 n = 6; EPA/Fx25 n = 6). Statistics: * indicate differences to CD and # indicates difference between Chrl and EPA/Fx diets5%, 15%, 25%. */# $p < 0.05$, **/## $p < 0.01$, ***/### $p < 0.001$. Abbreviations: OTUs, operational taxonomic unit's; further abbreviations see Figure 3.
